# Effect of Cryopreservation on Olive (*Olea europaea* L.) Plant Regeneration via Somatic Embryogenesis

**DOI:** 10.3390/plants10010034

**Published:** 2020-12-25

**Authors:** Fatiha Bradaï, Carolina Sánchez-Romero

**Affiliations:** Departamento de Botánica y Fisiología Vegetal, Campus de Teatinos s/n, Universidad de Málaga, 29071 Málaga, Spain; fatihar19@gmail.com

**Keywords:** embryogenic culture, genotype, morphogenic competence, regeneration capacity, somatic embryo

## Abstract

Olive somatic embryos have been successfully cryopreserved using the droplet-vitrification method on aluminum foil strips. Although acceptable recovery rates have been obtained after rewarming, the influence of this cryopreservation protocol on the somatic embryogenesis process is unknown. To evaluate the effect of cryopreservation on olive somatic embryogenesis, the behavior of cultures established from cryopreserved somatic embryos was compared with that of control, non-cryopreserved cultures in the different phases of the somatic embryogenesis process. In order to analyze the influence of the genotype, this investigation was carried out in two independent lines. During the proliferation step, only the line T1 was affected by cryopreservation, with higher fresh weight increases. Although similar total embryos were produced per culture, freezing in liquid nitrogen significantly improved the maturation pattern in the line P5. Better germination results were also found in this embryogenic line. The genotype plays a key role, largely determining the effect of cryopreservation on olive somatic embryogenesis. A specific genotype-dependent response was found depending on the culture step. Variations observed could not be associated to differences in the embryogenic lines’ instability to maintain their morphogenic competence after cryopreservation. Embryogenic cultures established after rewarming retained their regeneration capacity, with no evident negative effects affecting their regeneration capacity.

## 1. Introduction

The olive (*Olea europaea* L.) is the second most important oil fruit tree crop worldwide after oil palm. Its production has been traditionally concentrated in the Mediterranean basin, where this crop has a great impact on the economy, history, culture, and environment [1].

Somatic embryogenesis is a powerful in vitro technique, with multiple applications in plant breeding programs using both conventional or biotechnological means [2].

In olive, somatic embryogenesis was first reported by Rugini [3]. Since then, great improvement has been achieved and somatic embryogenesis protocols currently available allow the obtainment of an acceptable number of plants from embryogenic cultures initiated from mature zygotic embryos [4,5,6]. Thus, up to 8.30 somatic embryos gave rise to plantlets with shoots or shoots and roots per culture initiated in the maturation phase by using the protocol described by Sánchez-Romero [4,6].

Once established, embryogenic cultures require continuous maintenance through repetitive subcultures in the proliferation medium. However, long-term embryogenic culture maintenance is labor-intensive and space-consuming and represents a risk of tissue loss due to contamination, technical failure, or human error. Additionally, long-term proliferation provokes loss of embryogenic competence, thus compromising the regeneration ability of embryogenic cultures, and increasing the risk of occurrence of somaclonal variation [4,5].

Cryopreservation, i.e., the conservation of biological material at ultra-low temperatures, usually in liquid nitrogen (LN) [7], is a good alternative for the long-term conservation of embryogenic cultures. In fact, cryopreservation is considered the only technique currently available for safe and cost-effective long-term conservation of plant genetic resources [8]. At −196 °C, the temperature of LN, all metabolic processes are arrested and, therefore, plant material can be stored for a very long time [7].

Cryopreservation allows the long-term storage of valuable embryogenic lines used in propagation or breeding programs or utilized for application of biotechnological tools based in somatic embryogenesis systems. Apart from permitting the conservation and management of selected lines, it enables the preservation of transgenic material while field trials are on-going [9] or of the juvenile characteristics of clones until the results of progeny testing become available [10]. Moreover, it is useful for the safe long-term storage of plant tissues with specific characteristics, such as medicinal- and alkaloid-producing cell lines or hairy root cultures [11].

Both classical, based on slow cooling protocols, and new, vitrification-based methods have been used to conserve olive embryogenic cultures [12]. In 2017, Bradaï et al. [13] achieved successful cryopreservation of olive somatic embryos by using the droplet-vitrification method on aluminum foil strips. Using this procedure, 60% of samples resumed embryogenesis, thus ensuring the safe long-term conservation of this type of embryogenic structures.

However, during cryopreservation, embryogenic tissues are exposed to different processes, such as conditioning pretreatments, incubation in cryoprotectants solutions, cryostorage, and post-rewarming manipulations, which have a stressing effect and potentially may cause changes in their morphogenic capacity. Consequently, tissue regrowth after rewarming cannot be the only criterion for successful cryopreservation [14], and before incorporating cryopreservation into a biotechnology, conservation or breeding program, it is essential to elucidate its influence on the somatic embryogenesis process.

The objective of the present investigation was to evaluate the effect of cryopreservation on culture regeneration capacity, determining its effect on the efficiency of the different phases of the somatic embryogenesis process. Considering the influence of the genotype on embryogenic cultures behavior [4], the investigation was carried out in two independent embryogenic lines.

## 2. Results

### 2.1. Embryogenic Culture Proliferation

**Figure 1:**
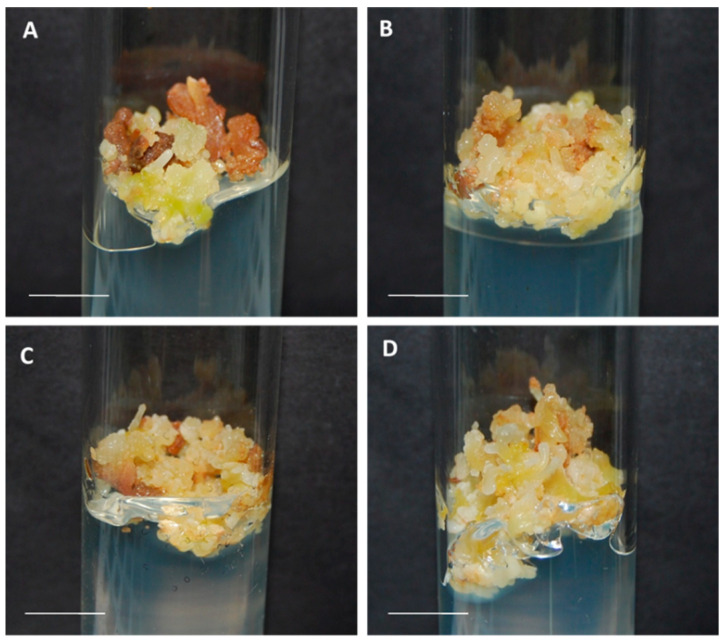
Appearance of olive embryogenic cultures (**A**,**C**) control and (**B**,**D**) derived from cryopreserved somatic embryos of the lines T1 (**A**,**B**) and P5 (**C**,**D**), six weeks after the last subculture on proliferation medium. Bar = 1 cm.

**Figure 2:**
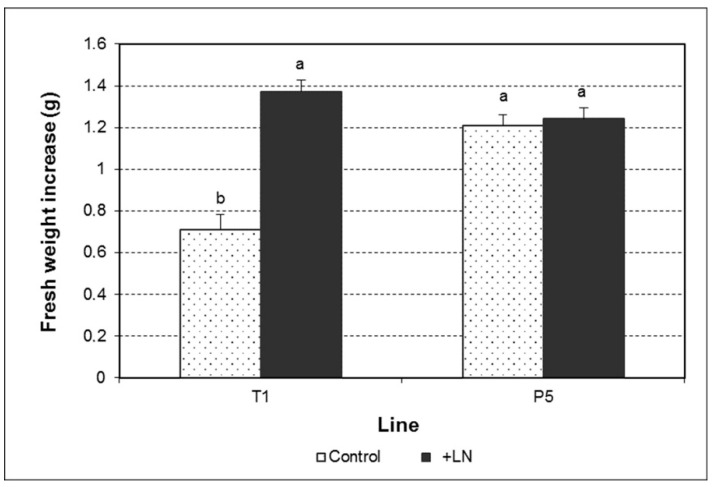
Fresh weight increase of olive embryogenic cultures control and derived from cryopreserved somatic embryos of the lines T1 and P5. Data assessed six weeks after the last subculture. Data represent the mean ± SEM. Different letters indicate significant differences by the least significant difference (LSD) test with a significance level of 0.05.

Control and cryopreserved cultures of each embryogenic line did not exhibit morphological differences during the proliferation phase (Figure 1). Nonetheless, fresh weight increase data revealed a significant effect of the cryopreservation, the genotype, and the interaction between both factors on this parameter (Figure 2; Table S1). Although cryopreservation did not modify proliferation of the line P5, higher fresh weight increases were found in the line T1 (Figure 2), with 0.71 g in control cultures versus 1.37 g in cultures derived from cryopreserved somatic embryos.

**Figure 3:**
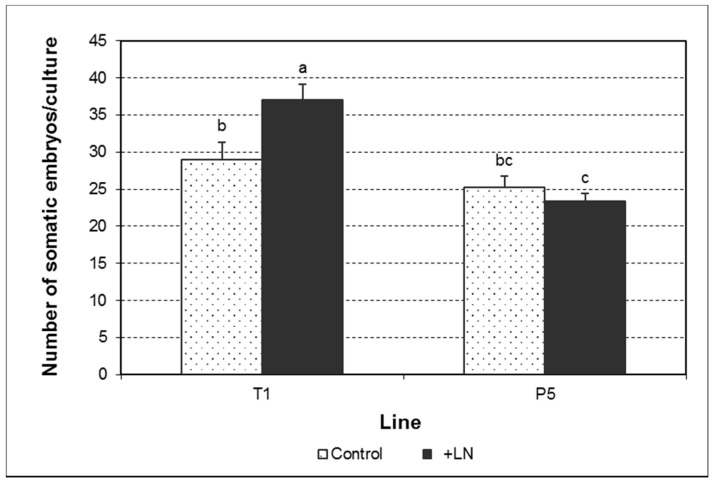
Number of somatic embryos developed per culture during the proliferation phase from control and cryopreserved embryogenic cultures of the lines T1 and P5. Data assessed six weeks after the last subculture. Data represent the mean ± SEM. Different letters indicate significant differences by the LSD test with a significance level of 0.05.

The production of somatic embryos in maintenance medium was significantly affected by the genotype and the interaction genotype × cryopreservation (Figure 3; Table S1). Thus, while a higher number of somatic embryos was found in the line T1 after cryopreservation, a slight decline was observed in the line P5. However, data regarding number of somatic embryos developed per gram of fresh weight revealed a significant decrease in culture capability to produce somatic embryos after cryopreservation (Table 1; Table S1). This effect was also influenced by the genotype and the interaction genotype × cryopreservation. Thus, control cultures of the line T1 yielded a higher number of embryos per gram of fresh weight, but the negative effects of cryostorage were more pronounced in this line than in P5. As shown in Table 1, the decrease in somatic embryo production observed in the line T1 was due to a decline in the development of translucent somatic embryos shorter than 5 mm (3–4 mm) (TrSE < 5). The production of translucent somatic embryos equal or larger than 5 mm (TrSE ≥ 5) and white-opaque somatic embryos shorter than 5 mm (WOSE < 5) only was determined by the genotype (Table S1). However, a clear effect of cryopreservation or the interaction genotype × cryopreservation on cultures proliferation pattern was not evident. Only the genotype determined the proportion of the different types of somatic embryos during the proliferation phase (Table 1; Table S1). Interestingly, cryopreservation only modified the proportion of WOSE < 5 in the line P5, which significantly increased from 3.31 to 7.36%.

### 2.2. Somatic Embryo Maturation

**Figure 4:**
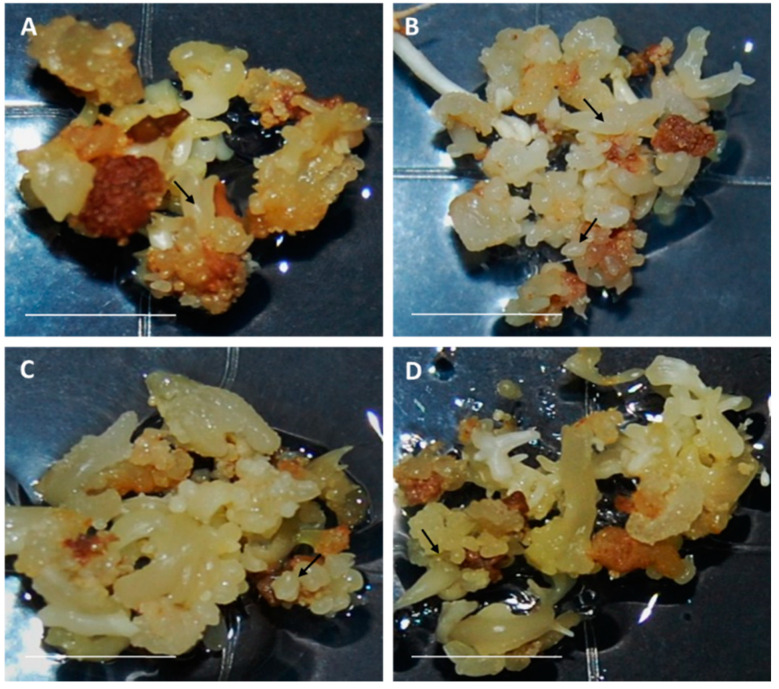
Appearance of olive embryogenic cultures (**A**,**C**) control and (**B**,**D**) derived from cryopreserved somatic embryos of the lines T1 (**A**,**B**) and P5 (**C**,**D**) after eight weeks in maturation medium. Arrows indicate somatic embryos. Bar = 1 cm.

**Figure 5:**
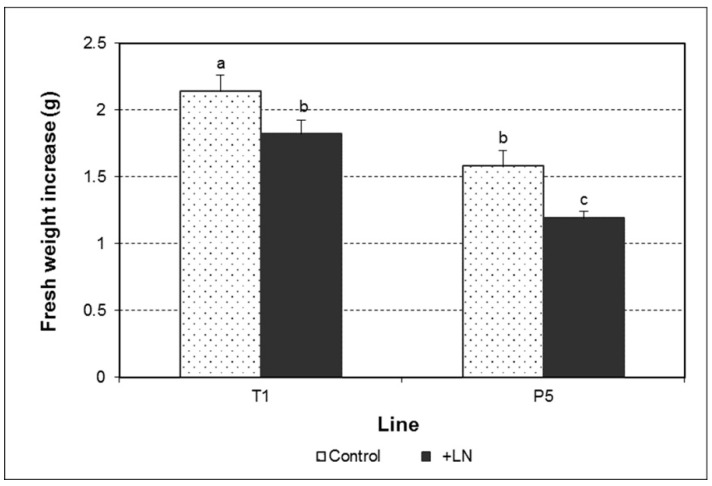
Fresh weight increase of olive embryogenic cultures control and derived from cryopreserved somatic embryos of the lines T1 and P5. Data assessed eight weeks after initiation in maturation conditions. Data represent the mean ± SEM. Different letters indicate significant differences by the LSD test with a significance level of 0.05.

**Figure 6:**
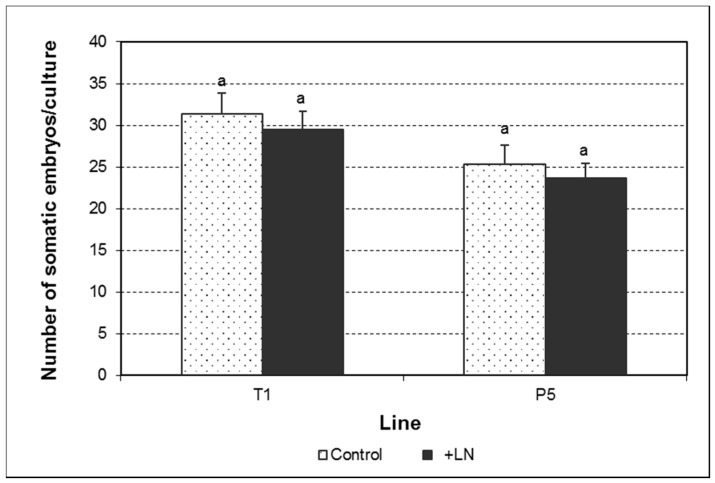
Number of somatic embryos developed per culture during the maturation phase from control and cryopreserved embryogenic cultures of the lines T1 and P5. Data assessed eight weeks after initiation in maturation conditions. Data represent the mean ± SEM. Different letters indicate significant differences by the LSD test with a significance level of 0.05.

The aspect of control and cryopreserved cultures did not show significant differences after eight weeks in maturation medium (Figure 4). Fresh weight increase during the maturation step significantly decreased after cryopreservation (Figure 5; Table S2). A slight decline in the total production of somatic embryos was evident after this process (Figure 6), although only the genotype exhibited a significant influence on this variable (Table S2).

Although, as revealed by the number of total somatic embryos produced per gram of culture, the ability of embryogenic cultures to form somatic embryos did not change due to cryopreservation, a significant effect of the genotype, cryopreservation, and interaction genotype × cryopreservation was found in TrSE ≥ 5 mm (Table 2; Table S2). While in the line P5 cryopreservation provoked a significant increase of this type of embryo, from 5.19 to 8.77 embryos/gram, in the line T1, only a slight increment could be observed, from 3.69 to 3.88 embryos/gram of culture. The ability to produce somatic embryos at the rest of the developmental stages was not influenced by any predictor variable (Table S2). Cryopreservation significantly altered culture structure in the line P5, with an increase in the proportion of TrSE ≥ 5 produced (Table 2; Table S2). This variation was accompanied by a decline in the percentage of embryos at earlier developmental stages (TrSE < 5) (Table 2). No modifications in the maturation pattern were evident in the line T1.

### 2.3. Somatic Embryo Germination

**Figure 7:**
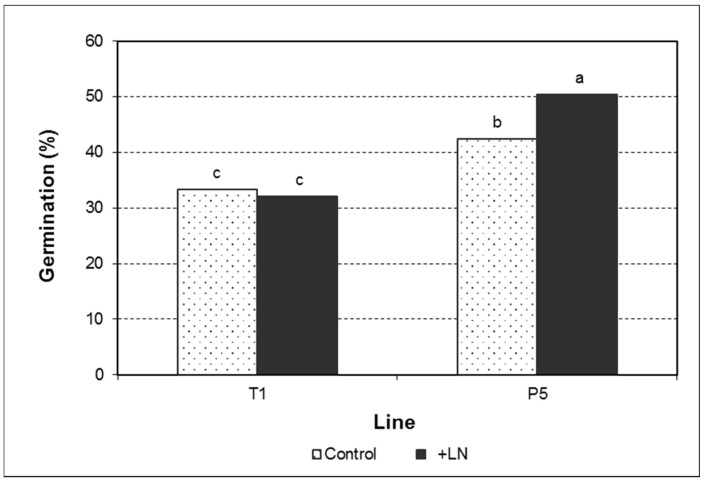
Germination of olive somatic embryos developed from control and cryopreserved embryogenic cultures of the lines T1 and P5. Data assessed after two six-week recultures on germination conditions. Different letters indicate significant differences by the R × C test of independence with a significance level of 0.05.

Germination of somatic embryos was significantly affected by the genotype, cryopreservation, and the genotype × cryopreservation interaction (Figure 7; Table S3). Nevertheless, different influences were observed depending on the embryo developmental stage (Table 3; Table S3). Thus, while in TrSE < 5 and TrSE ≥ 5 germination frequency was determined by the genotype, in WOSE < 5, cryopreservation was the main predictor variable. A significant effect of the cryopreservation and the interaction genotype × cryopreservation was found in TrSE < 5.

**Figure 8:**
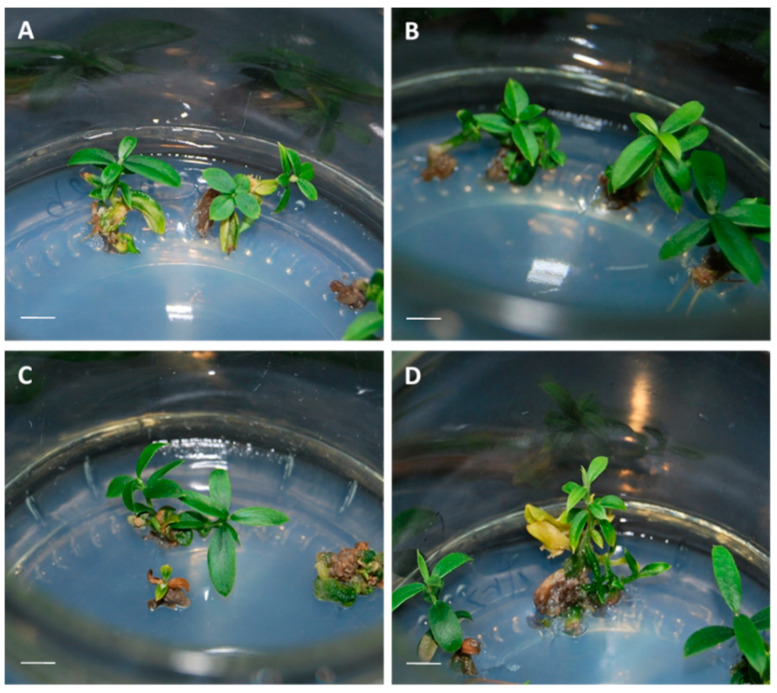
Germination of olive somatic embryos developed from control (**A**,**C**) and cryopreserved (**B**,**D**) cultures of the lines T1 (**A**,**B**) and P5 (**C**,**D**). Bar = 1 cm.

In the line T1, cryopreservation did not influence embryo germination (Figure 7). No effects on the germination of the different types of embryos could be observed either (Table 3). However, in the line P5, embryos derived from cryopreserved cultures exhibited higher germination capacity than those developed from control, non-frozen cultures (Figure 7). This positive effect was found in all types of embryos, independently of their developmental stage, although no significant differences were evident in TrSE ≥ 5 mm (Table 3, Table S3). Plantlets exhibited a good aspect in all cases (Figure 8).

## 3. Discussion

Five to six months after rewarming, cultures established from somatic embryos cryopreserved following the protocol of Bradaï et al. [13] presented a good aspect. Although in general, higher proliferation rates were found in cryopreserved lines compared to control, non-frozen cultures; as found in previous investigations [14,15], the genotype exerted a primary effect, determining the significance of the influence. Higher proliferation rates after cryopreservation have been reported in different somatic embryogenesis systems, such as *Hevea brasiliensis* [16], *Castanea dentata* [17], *Araucaria angustifolia* [18], *Pinus nigra* [14], or *Abies cephalonica* [19]. Nevertheless, no influence or a negative effect of cryopreservation on culture proliferation have also been reported in *Picea abies* [15], *Persea americana* [20], and *Vitis vinifera* [21], where slower regrowth and lower fresh weight increases were found after this process.

Most investigations reported maintenance of morphological appearance and growth pattern after cryopreservation [14,21,22] or increased synchronization and better development of somatic embryos [17,23]. However, cryopreservation influenced the proliferation pattern of olive embryogenic cultures in a genotype-dependent manner. Although the total number of somatic embryos produced per culture increased in the line T1, culture quality decreased, as fewer somatic embryos were obtained per gram fresh weight. This effect was mainly due to a significant decrease in the development of TrSE < 5 per gram of culture, the type of embryos mostly found in this culture phase. On the contrary, in the line P5, no significant changes were observed in this sense, although a significant increase in the proportion of WOSE < 5 was found in this line.

In relation to the maturation phase, no significant influence of cryopreservation was found in olive embryogenic cultures. Although similar results were reported in *Gentiana* spp. [22] and *Gentiana cruciata* [24], investigations involving several cell lines revealed a significant effect of the genotype, with some embryogenic lines yielding the same number of mature somatic embryos than non-frozen cultures, whereas other lines produced a slightly lower number of mature somatic embryos than controls [19,25,26].

In relation to the culture developmental pattern, a significant impact of cryopreservation was observed in the line P5, with increased production of TrSE ≥ 5, both proportionally and per gram of culture. Number of TrSE ≥ 5 increased at the expense of TrSE < 5, thus revealing an effect of cryopreservation inducing embryo development to more advanced stages. In *Abies cephalonica*, however, Krajňáková et al. [19] reported a similar developmental pathway from pre-cotyledonary and cotyledonary somatic embryos before and after a long-term cryostorage period.

The effect of cryopreservation on germination capacity of olive somatic embryos varied significantly depending on the genotype. Thus, while in the line P5 a significant positive effect was found; in the line T1, no variation could be observed. In *Quercus suber*, Valladares et al. [27] obtained similar germination rates in cryopreserved embryos compared to control embryos. However, Fang et al. [28] observed a decline in the germination capacity of cocoa embryos after cryopreservation. Increase in the germination ability observed in olive somatic embryos derived from cryopreserved cultures may evince improvement of the differentiation process, as germination depends on the correct histodifferentation and reserve products accumulation occurring during embryo development and maturation [4]. These results concur with those obtained in the maturation phase, in which improvement of culture structure was found in the line P5 after cryopreservation.

Different causes may justify the effects of cryopreservation on somatic embryogenesis. Higher morphogenic potential after cryopreservation has been explained by a differential response of embryogenic and non-embryogenic cells to this technique [29]. Hence, cryopreservation may act as a selective process, leading to cultures enriched in embryogenic cells [17]. Positive effects of cryopreservation have also been related to increased synchronization of development from embryogenic cells [14,30,31].

According to Barra-Jiménez et al. [32], alteration of the differentiation ability of embryogenic cultures after cryopreservation may also have an epigenetic and genetic basis. Alterations at gene transcription level, such as DNA methylation or histone modification, have been observed in different steps of the cryopreservation procedure [33], such as the preculture phase [34] and the treatment with the vitrification solution [35]. Microsatellites analysis showed genetic alterations during the cryopreservation process [32]. In fact, the yield of cotyledonary embryos has been positively correlated with genetic stability [32,36].

The present investigation reveals a primary role of the genotype on culture response to cryopreservation. Differences among genotypes could be due to differences in the nature of embryogenic tissues related to the proportion of hyperhydric vacuolated cells within them [37], to the physiological conditions of tissues [38], or to the physiological response triggered by cryopreservation, which may involve the activity of specific cell wall-plasma membrane proteins [39] or the activation of enzymes protecting against oxidative stress [40].

Differences in tissues stability have also been related to differences in their genetic make-up whereby some components of the genome make it more prone to display variation [41]. Furthermore, as epigenetic changes could be affected by the individual genetic endowment [41], different genotypes may also undergo different epigenetic modifications in response to stress during cryopreservation.

In conclusion, the genotype plays a key role largely determining the effect of cryopreservation on olive somatic embryogenesis. A specific genotype-dependent response was found depending on the culture step. Thus, while in the proliferation phase significant variations were only observed in the line T1; in the maturation and germination steps, only the line P5 was affected by cryopreservation. Variations observed could not be associated to differences in the embryogenic lines’ instability to maintain their morphogenic competence after cryopreservation. Nevertheless, embryogenic cultures established after rewarming retain their morphogenic capacity for subsequent regeneration of plants, with no evident negative effects affecting the somatic embryogenesis process executed following the procedure described by Sánchez-Romero [6]. Therefore, the droplet-vitrification method optimized by Bradaï et al. [13] can be considered a reliable cryopreservation procedure for the long-term conservation of olive embryogenic lines.

## 4. Materials and Methods

### 4.1. Plant Material and Culture Conditions

Olive (*Olea europaea* L.) embryogenic lines were initiated from radicle of mature zygotic embryos “Picual” following the protocol of Orinos and Mitrakos [42]. Radicle segments were cultured for three weeks on OMc medium [43] supplemented with 25 μM indole-3-butyric acid (IBA) and 2.5 μM 2-isopentenyl-adenine (2iP) and subsequently transferred to basal OMc medium with 2.5 μM IBA until development of embryogenic callus.

Embryogenic cultures obtained from single zygotic embryos were maintained as independent lines by repetitive embryogenesis on olive cyclic embryogenesis (ECO) medium [44,45]. Subcultures of the embryogenic cell lines were performed at six–seven-week intervals. All cultures were incubated in darkness at 25 ± 1 °C.

The pH of the media was adjusted to 5.74 before adding the gelling agent, consisting of agar 6 g L^−1^, except for ECO medium, which was gelled with 3 g L^−1^ phytagel. Media sterilization was carried out by autoclaving for 20 min at 121°C and 0.1 MPa.

### 4.2. Cryopreservation Procedure

Cryopreservation of olive somatic embryos was carried out following the protocol of Bradaï et al. [13], using the droplet vitrification method on aluminum foil strips [46]. Briefly, somatic embryos 1–6 mm in size were selected from stock cultures of two independent embryogenic lines (T1 and P5) at the end of a maintenance cycle. One hundred milligrams of somatic embryos (8 to 12 embryos) were incubated in 10 mL of loading solution (LS) sterilized by filtration. LS solution consisted of 2 M glycerol and 0.4 M sucrose dissolved in basal ECO medium (pH 5.74). After 20 min in darkness and at room temperature, LS solution was substituted for approximately 10 mL of PVS2 solution at 0°C, composed of 3.26 M glycerol, 2.42 M ethylene glycol, 1.9 M dimethyl sulfoxide, and 0.4 M sucrose in basal ECO medium (pH 5.74). Explants surrounded by a droplet of PVS2 solution were placed on aluminum foil strips. In order to keep the temperature of aluminum strips at 0 °C, all manipulations were executed on frozen cooling plates. Following PVS2 exposure for 30 min, the aluminum strips were plunged in LN for at least 30 min. For rewarming, aluminum strips were taken out from LN and quickly submerged in recovery solution, consisting of basal ECO medium with 1.2 M sucrose (pH 5.74). Incubation in recovery solution was carried out at room temperature for 15 min. Subsequently, somatic embryos were placed onto two discs of sterile filter paper disposed on ECO medium containing 0.3 M sucrose and 0.001% (*w*/*v*) ascorbic acid. Twenty-four hours later, cultures were transferred to ECO standard proliferation medium [4] and incubated in darkness at 25 ± 1 °C.

### 4.3. Effect of Cryopreservation on Somatic Embryogenesis

In order to investigate the effect of cryopreservation on somatic embryogenesis, the behavior of cultures established from cryopreserved somatic embryos was compared with that of control, non-cryopreserved cultures in the different phases of the somatic embryogenesis process, five to six months after rewarming. To examine the effect of the genotype, control and cryopreserved cultures of two independent embryogenic lines (T1 and P5) were examined during the proliferation, maturation, and germination steps.

For evaluation of culture proliferation, 200 mg of embryogenic tissue were selected from control and cryopreserved stock cultures six weeks after the last subculture and inoculated in 25 × 150 mm test tubes containing 25 mL of ECO medium. After a six-week culture cycle at 25 ± 1 °C in darkness, culture aspect, fresh weight increase, and number of somatic embryos at different developmental stages (TrSE < 5, TrSE ≥ 5, WOSE < 5 and white-opaque somatic embryos equal or larger than 5 mm (WOSE ≥ 5)) [4] were assessed.

Somatic embryo development was carried out by culturing 100 mg of embryogenic tissue in 90 × 25 mm Petri dishes containing 50 mL of basal ECO medium, i.e., ECO medium lacking plant growth regulators and cefotaxime. Eight weeks after incubation at 25 ± 1 °C and darkness, culture appearance, fresh weight increase, and number of TrSE < 5 mm, TrSE ≥ 5 mm, WOSE < 5 mm, and WOSE ≥ 5 mm were recorded.

Somatic embryos equal or larger than 3 mm derived from maturation conditions were individually cultivated in 85 × 80 mm jars containing 50 mL of Clavero-Ramírez and Pliego-Alfaro [47] medium for inducing germination. Germination was carried out at 25 ± 1 °C under a 16 h photoperiod and 40 μmol m^−2^ s^−1^ irradiance provided by Grolux lamps (Sylvania, Erlangen, Germany). Germination percentage was recorded after two recultures of six weeks each.

### 4.4. Data Taken and Statistical Analysis

In the proliferation and maturation phases, 20 cultures were used per treatment and embryogenic line. The number of somatic embryos included at each repetition of the germination experiment ranged from 298 to 547, depending on the performance of the previous maturation phase, uneven for the different treatments and embryogenic lines. All experiments were repeated twice. Somatic embryos were considered germinated when shoot and/or root elongation was equal or larger than 2 mm.

Percentage data were analyzed with an R × C test of independence or a three-way log-linear analysis, using the BIOMstat software (Exeter Software, Setauket, NY, USA). The rest of the data were analyzed by one- or two-way ANOVA followed by mean comparison by the LSD test, using the software package SPSS 26.0 (IBM SPSS Statistics, USA). The significance level used was 0.05 in all cases [48].

## Figures and Tables

**Table 1:** Effect of cryopreservation on the capability of embryogenic cultures of the lines T1 and P5 to produce somatic embryos at different developmental stages during the proliferation phase. Production of somatic embryos at different developmental stages expressed per gram of culture and proportionally with respect to the total number of embryos obtained. Data assessed six weeks after the last subculture. Different letters indicate significant differences by the LSD test with a significance level of 0.05.

Embryogenic Line	Cryopreservation	Number of Somatic Embryos per g of Culture					Proportion (%) of Somatic Embryos			
		TrSE < 5	TrSE ≥ 5	WOSE < 5	WOSE ≥ 5	Total	TrSE < 5	TrSE ≥ 5	WOSE < 5	WOSE ≥ 5
T1	Control	42.87 a	3.48 b	0.26 b	0.00 a	46.61 a	90.30 a	8.87 b	0.83 b	0.00 a
	+LN	24.04 b	3.97 b	0.37 b	0.05 a	28.43 b	83.24 a	15.39 b	1.19 b	0.17 a
P5	Control	14.38 c	6.72 a	0.77 ab	0.12 a	21.98 bc	63.55 b	32.38 a	3.31 b	0.76 a
	+LN	12.65 c	4.86 ab	1.36 a	0.23 a	19.10 c	65.73 b	25.73 a	7.36 a	1.18 a

LN: liquid nitrogen; TrSE < 5: translucent somatic embryos shorter than 5 mm (3–4 mm); TrSE ≥ 5: translucent somatic embryos equal or larger than 5 mm; WOSE < 5: white-opaque somatic embryos shorter than 5 mm (3–4 mm); WOSE ≥ 5: white-opaque somatic embryos equal or larger than 5 mm.

**Table 2:** Effect of cryopreservation on the capability of embryogenic cultures of the lines T1 and P5 to produce somatic embryos at different developmental stages during the maturation phase. Production of somatic embryos at different developmental stages expressed per gram of culture and proportionally with respect to the total number of embryos obtained. Data assessed eight weeks after initiation in maturation conditions. Different letters indicate significant differences by the LSD test with a significance level of 0.05.

Embryogenic Line	Cryopreservation	Number of Somatic Embryos per g of Culture					Proportion (%) of Somatic Embryos			
		TrSE < 5	TrSE ≥ 5	WOSE < 5	WOSE ≥ 5	Total	TrSE < 5	TrSE ≥ 5	WOSE < 5	WOSE ≥ 5
T1	Control	10.73 a	3.69 c	0.73 a	0.35 a	15.50 a	68.23 a	24.65 c	4.50 a	2.63 a
	+LN	12.65 a	3.88 bc	0.58 a	0.33 a	17.44 a	71.21 a	23.33 c	3.57 a	1.89 a
P5	Control	11.10 a	5.19 b	0.62 a	0.46 a	17.37 a	60.46 b	33.59 b	3.62 a	2.33 a
	+LN	10.84 a	8.77 a	0.62 a	0.65 a	20.89 a	48.50 c	45.77 a	2.65 a	3.08 a

LN: liquid nitrogen; TrSE < 5: translucent somatic embryos shorter than 5 mm (3–4 mm); TrSE ≥ 5: translucent somatic embryos equal or larger than 5 mm; WOSE < 5: white-opaque somatic embryos shorter than 5 mm (3–4 mm); WOSE ≥ 5: white-opaque somatic embryos equal or larger than 5 mm.

**Table 3:** Germination percentages of somatic embryos developed from control and cryopreserved embryogenic cultures of the lines Table 1. and P5. Data assessed after two six-week recultures in the germination. Different letters indicate significant differences by the R × C test of independence with a significance level of 0.05.

Embryogenic Line	Cryopreservation	Number of SE at Different Developmental Stages				Global
		TrSE < 5	TrSE ≥ 5	WOSE < 5	WOSE ≥ 5	
T1	Control	33.82 c	29.25b	48.65 ab	36.00 b	33.30 c
	+LN	31.18 c	29.69 b	65.63 a	38.46 b	32.05 c
P5	Control	45.20 b	36.71 ab	38.46 b	39.13 b	42.39 b
	+LN	54.38 a	43.26 a	68.00 a	70.00 a	50.34 a

LN: liquid nitrogen; TrSE < 5: translucent somatic embryos shorter than 5 mm (3–4 mm); TrSE ≥ 5: translucent somatic embryos equal or larger than 5 mm; WOSE < 5: white-opaque somatic embryos shorter than 5 mm (3–4 mm); WOSE ≥ 5: white-opaque somatic embryos equal or larger than 5 mm.

## Supplementary Materials

The following are available online at https://www.mdpi.com/2223-7747/10/1/34/s1, Table S1, Table S2 and Table S3.
