# Supplementary material for: Effect of Cryopreservation on Olive (Olea europaea L.) Plant Regeneration via Somatic Embryogenesis
Source: Plants (Basel). 2020 Dec 25;10(1):34. doi: 10.3390/plants10010034 (PMC7823602; doi:10.3390/plants10010034)
Supplement: Supplementary file 1 [file plants-10-00034-s001.zip › Table S1_Proofs.docx]

**Table S1.** Significance by two-way ANOVA of single and combined effects of cryopreservation and genotype for the parameters determined during the proliferation phase.

| **Predictor variable** | **Fresh weight increase** | **Number of somatic embryos** | **Number of somatic embryos per g of culture** | | | | |  |  | | **Proportion (%) of somatic embryos** | | | | |  |
| --- | --- | --- | --- | --- | --- | --- | --- | --- | --- | --- | --- | --- | --- | --- | --- | --- |
|  |  |  | **TrSE<5** | **TrSE≥5** | **WOSE<5** | **WOSE≥5** | **Total** | | |  | | **TrSE<5** | **TrSE≥5** | **WOSE<5** | **WOSE≥5** | |
| Genotype | 0.005 | 0.000 | 0.000 | 0.023 | 0.010 | 0.074 | 0.000 | | |  | | 0.000 | 0.000 | 0.001 | 0.048 | |
| Cryopreservation | 0.000 | 0.115 | 0.001 | 0.441 | 0.223 | 0.303 | 0.001 | | |  | | 0.498 | 0.985 | 0.078 | 0.498 | |
| Genotype x Cryopreservation | 0.000 | 0.013 | 0.005 | 0.190 | 0.399 | 0.723 | 0.011 | | |  | | 0.202 | 0.051 | 0139 | 0.778 | |

LN: liquid nitrogen; TrSE<5: translucent somatic embryos shorter than 5 mm (3–4 mm); TrSE≥5: translucent somatic embryos equal or larger than 5 mm; WOSE<5: white-opaque somatic embryos shorter than 5 mm (3–4 mm); WOSE≥5: white-opaque somatic embryos equal or larger than 5 mm.
