# Supplementary material for: Effect of Cryopreservation on Olive (Olea europaea L.) Plant Regeneration via Somatic Embryogenesis
Source: Plants (Basel). 2020 Dec 25;10(1):34. doi: 10.3390/plants10010034 (PMC7823602; doi:10.3390/plants10010034)
Supplement: Supplementary file 1 [file plants-10-00034-s001.zip › Table S2_Proofs.docx]

**Table S2.** Significance by two-way ANOVA of single and combined effects of cryopreservation and genotype for the parameters determined during the maturation phase.

| **Predictor variable** | **Fresh weight increase** | **Number of somatic embryos** | **Number of somatic embryos per g of culture** | | | | |  |  | | **Proportion (%) of somatic embryos** | | | | |  |
| --- | --- | --- | --- | --- | --- | --- | --- | --- | --- | --- | --- | --- | --- | --- | --- | --- |
|  |  |  | **TrSE<5** | **TrSE≥5** | **WOSE<5** | **WOSE≥5** | **Total** | | |  | | **TrSE<5** | **TrSE≥5** | **WOSE<5** | **WOSE≥5** | |
| Genotype | 0.000 | 0.011 | 0.541 | 0.000 | 0.839 | 0.086 | 0.074 | | |  | | 0.000 | 0.000 | 0.372 | 0.506 | |
| Cryopreservation | 0.001 | 0.439 | 0.477 | 0.000 | 0.667 | 0.483 | 0.067 | | |  | | 0.063 | 0.026 | 0.346 | 0.997 | |
| Genotype x Cryopreservation | 0.735 | 0.969 | 0.352 | 0.001 | 0.638 | 0.373 | 0.592 | | |  | | 0.002 | 0.006 | 0.984 | 0.268 | |

LN: liquid nitrogen; TrSE<5: translucent somatic embryos shorter than 5 mm (3–4 mm); TrSE≥5: translucent somatic embryos equal or larger than 5 mm; WOSE<5: white-opaque somatic embryos shorter than 5 mm (3–4 mm); WOSE≥5: white-opaque somatic embryos equal or larger than 5 mm.
