# Supplementary material for: Effect of Cryopreservation on Olive (Olea europaea L.) Plant Regeneration via Somatic Embryogenesis
Source: Plants (Basel). 2020 Dec 25;10(1):34. doi: 10.3390/plants10010034 (PMC7823602; doi:10.3390/plants10010034)
Supplement: Supplementary file 1 [file plants-10-00034-s001.zip › Table S3_Proofs.docx]

**Table S3.** Significance by three-way log-linear analysis of single and combined effects of cryopreservation and genotype for the parameters determined during the germination phase.

| **Predictor variable** | **Germination (%)** | | | | |
| --- | --- | --- | --- | --- | --- |
|  | **TrSE<5** | **TrSE≥5** | **WOSE<5** | **WOSE≥5** | **Total** |
| Genotype | 0.000 | 0.001 | 0.657 | 0.151 | 0.000 |
| Cryopreservation | 0.011 | 0.297 | 0.024 | 0.077 | 0.005 |
| Genotype x Cryopreservation | 0.005 | 0.345 | 0.475 | 0.194 | 0.006 |

LN: liquid nitrogen; TrSE<5: translucent somatic embryos shorter than 5 mm (3–4 mm); TrSE≥5: translucent somatic embryos equal or larger than 5 mm; WOSE<5: white-opaque somatic embryos shorter than 5 mm (3–4 mm); WOSE≥5: white-opaque somatic embryos equal or larger than 5 mm.
